# A Case of Achieving Urinary Continence Using Gracilis Muscle Flap for Recanalization and Fistula After Bladder Neck Closure

**DOI:** 10.1002/iju5.70012

**Published:** 2025-03-26

**Authors:** Naoki Wada, Haruka Takagi, Taichiro Ishimaru, Daiki Kikuchi, Miyu Ohtani, Hidehiro Kakizaki, Takeshi Yamao, Toshihiko Hayashi

**Affiliations:** ^1^ Department of Renal and Urologic Surgery Asahikawa Medical University Asahikawa Japan; ^2^ Department of Plastic and Reconstructive Surgery Asahikawa Medical University Asahikawa Japan

**Keywords:** bladder neck closure, gracilis muscle flap, spina bifida, urinary continence

## Abstract

**Introduction:**

We present the case of a patient who achieved urinary continence after undergoing a gracilis muscle flap (GMF) for recanalization and fistula after bladder neck closure (BNC).

**Case Presentation:**

A male patient with neurogenic lower urinary tract dysfunction complained of difficulty inserting the catheter because of numerous false cavities in the prostatic urethra. We performed BNC to create a continent catheterizable stoma. However, the bladder neck was recanalized in the early postoperative period. Next, we dissected and closed the urethra transperineally. Due to the tiny fistula, the patient's urinary incontinence persisted. Finally, we dissected and closed the urethra again transperineally, and the closed area was covered with a GMF. Since then, the urethra has been completely closed, and urinary continence has been achieved.

**Conclusion:**

The possibility of recanalization after BNC must be kept in mind. Interposition and covering of the GMF are useful techniques for urethral and perineal fistulas.

**Abbreviations**
BNC: bladder neck closure
GMF: gracilis muscle flap
NLUTD: neurogenic lower urinary tract dysfunction
UTI: urinary tract infection

**Summary**

- Although bladder neck closure (BNC) for intractable urinary incontinence is an effective last resort, the possibility of recanalization must be kept in mind.
- Interposition and covering of the gracilis muscle flap (GMF) are powerful and useful techniques for urethral and perineal fistulas.

## 1 Introduction

Bladder neck closure (BNC) is an effective procedure for achieving continence in complex cases with intractable urinary incontinence [1]. However, a well‐known potential complication following BNC is vesicoureteral recanalization or fistula. Reportedly, the incidence of recanalization or fistula ranges from 8% to 40% [2, 3]. Gracilis muscle flap (GMF) is often used to repair urinary fistulas such as rectourethral fistulas [4]. Herein, we present the case of a patient in whom urinary continence was achieved using GMF for recanalization and fistula after BNC and urethral dissection.

## 2 Case Report

**FIGURE 1:**
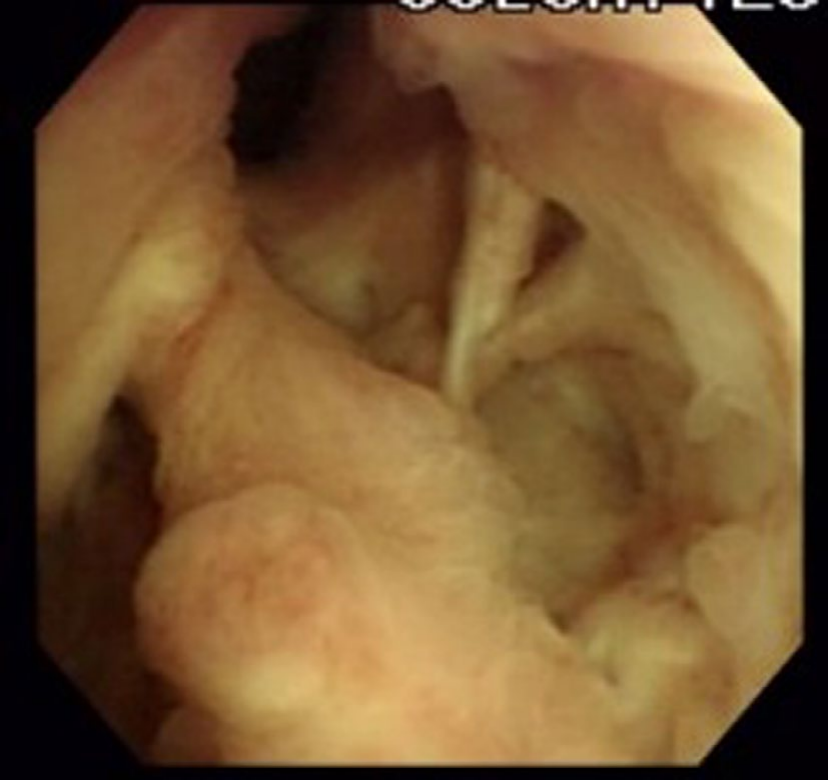
Cystourethroscopy revealed the formation of numerous false cavities in the prostatic urethra, which made it difficult to insert catheters.

The patient was a man in his 50s with neurogenic lower urinary tract dysfunction (NLUTD) due to spina bifida who used a wheelchair for daily living. Because he had developed recurrent febrile urinary tract infections (UTIs) at some point, he changed the type of catheter equipment or used indwelling balloon catheters during the night. Subsequently, he complained of difficulty inserting the catheter and worsening urinary incontinence. Cystourethroscopy revealed the formation of numerous false cavities in the prostatic urethra (Figure 1). Videourodynamics showed no evidence of detrusor overactivity or decreased bladder compliance with anticholinergic and β3 agonist treatment, and the abdominal leak point pressure was 35 cmH_2_O. We planned to perform the BNC and create a continent catheterizable stoma to the umbilicus without augmentation cystoplasty. Because there was not enough great omentum to intervene, the bladder neck was simply dissected and closed. We created the catheterizable stoma using the spiral Monti procedure [5] because of the unavailable obstructed appendix. However, cystography on postoperative day 22 revealed the recanalized bladder neck, and urinary incontinence persisted.

**FIGURE 2:**
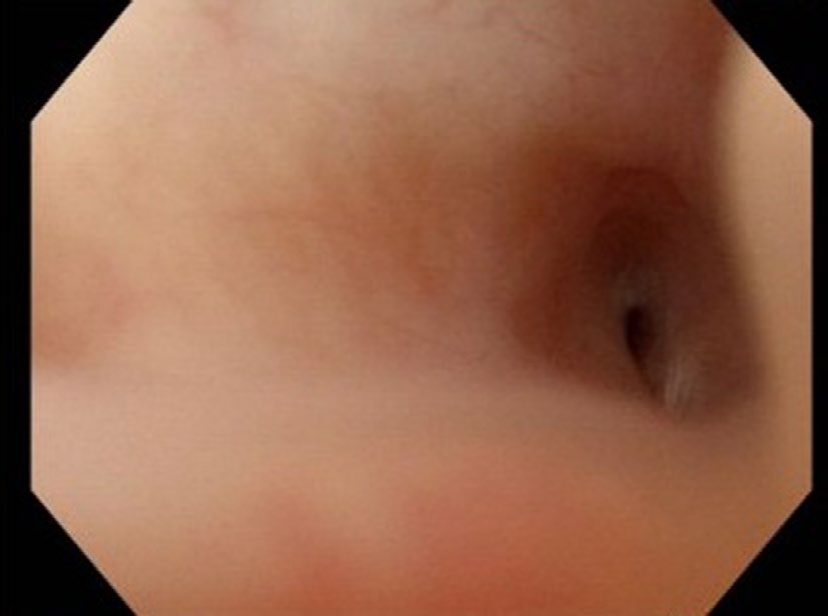
Urethroscopy revealed the tiny fistula at the dissected urethra.

The next step was to dissect and close the urethra transperineally. We dissected the urethra on the proximal side of the bulbourethra and closed both the proximal and distal ends of the urethra by covering them with the subcutaneous tissue of the scrotum. However, cystography on postoperative day 15 showed a tiny fistula. Thus, urinary incontinence did not improve due to a tiny fistula connecting the urethra and bladder at the dissected urethra (Figure 2).

**FIGURE 3:**
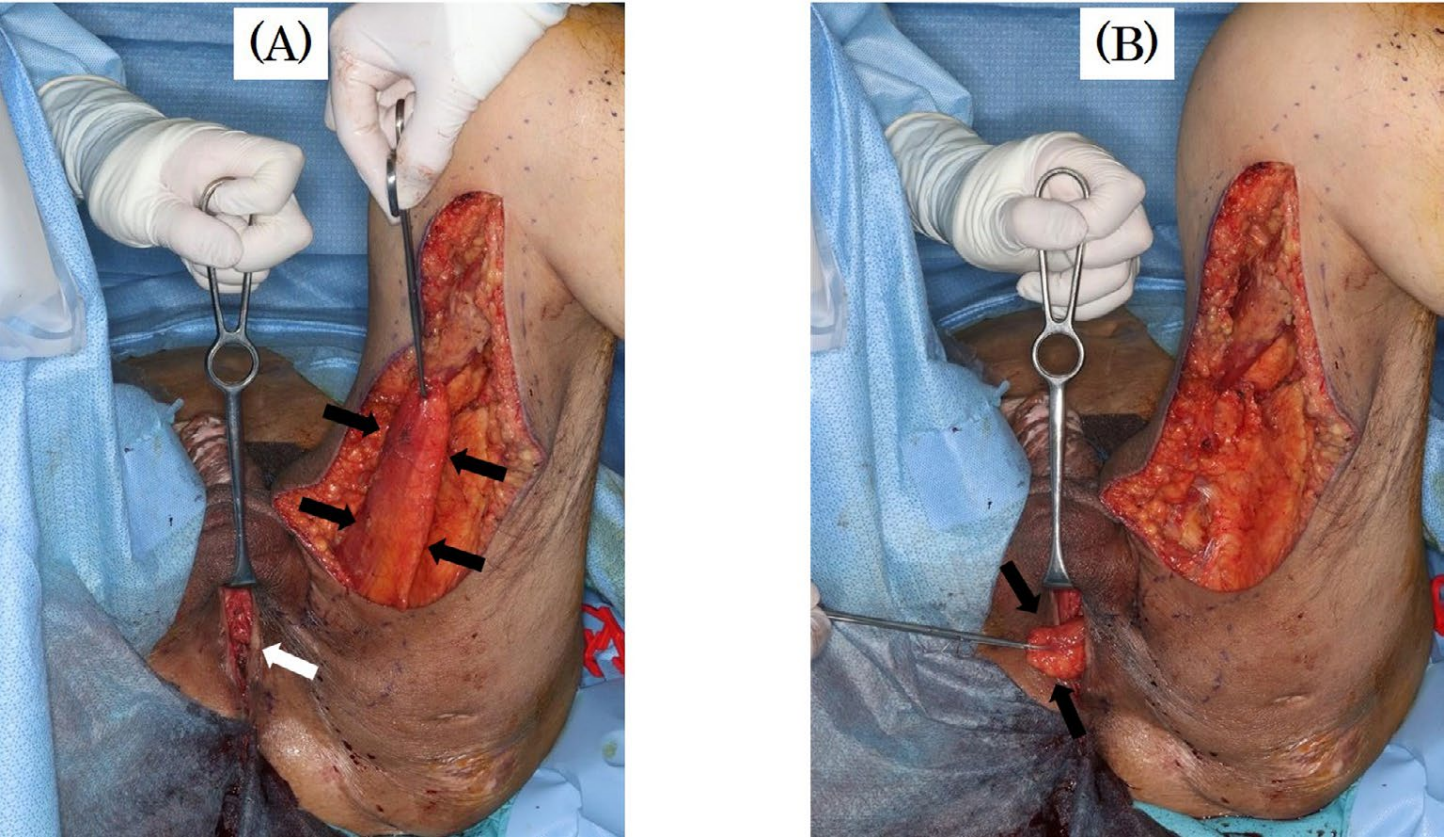
Tthe left gracilis muscle flap was harvested (black arrows), and white arrows indicated the opened wound of the perineum with dissection and closure of the urethra (A). The pedicled muscle flap (black arrows) was filled in the space of the perineum (B).

**FIGURE 4:**
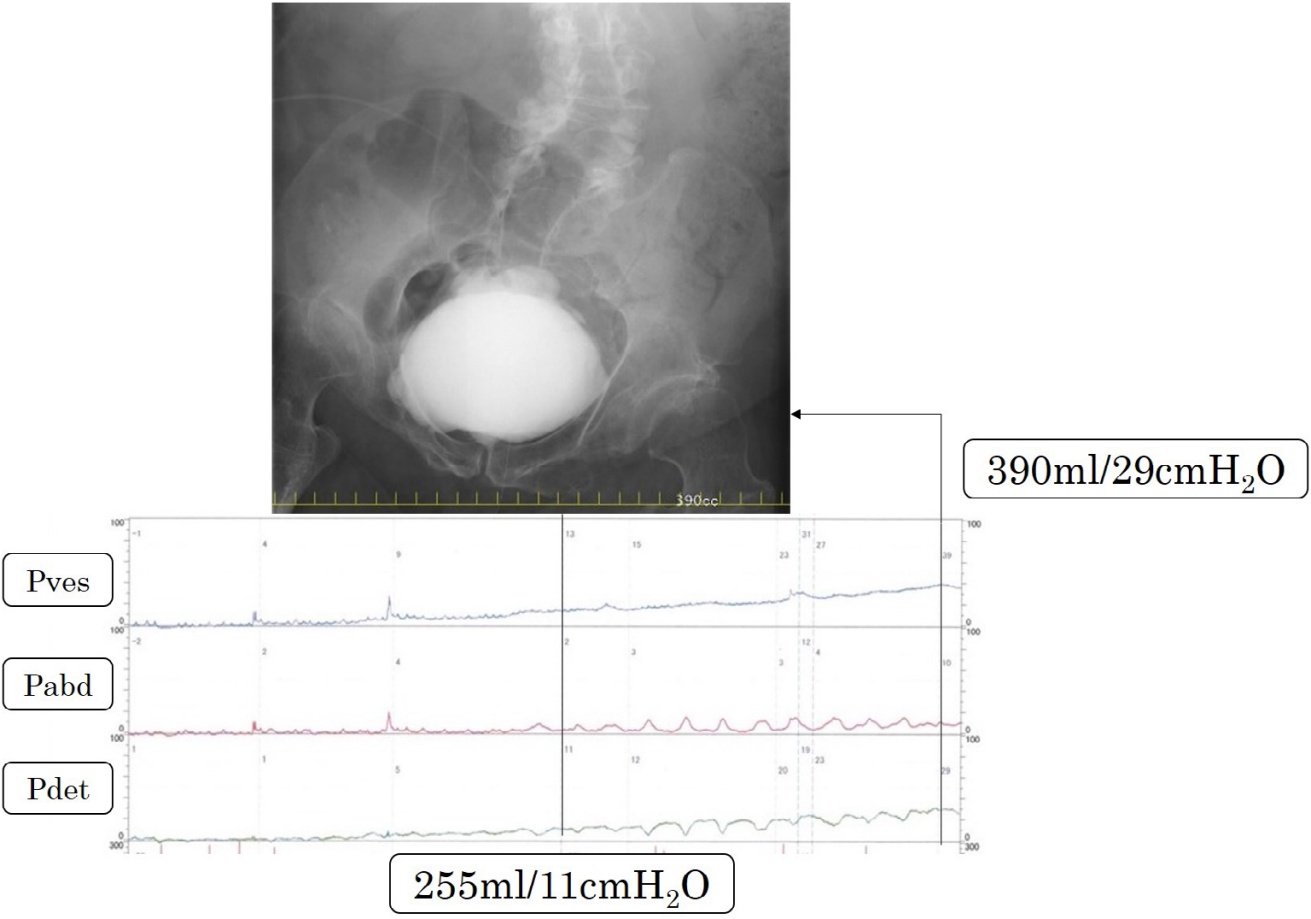
The most recent videourodynamics shows mild deformity of the bladder and increased detrusor pressure, but no vesicoureteral reflux. The detrusor pressure was 11 and 29 cmH2O at 255 and 390 mL bladder capacity, respectively (bladder compliance: 390 mL/29 cmH_2_O = 13 mL/cmH_2_O).

Finally, we dissected the urethra and closed it again transperineally, followed by re‐resection of the bulbourethra. Because the Department of Plastic and Reconstructive Surgery was established in our hospital after the patient's second surgery, we referred the patient to plastic surgeons to cover the closed area with a GMF (Figure 3). He developed cellulitis of the muscle flap–harvested wound, but since then, the urethra has been completely closed and urinary continence was achieved. Videourodynamics at 1 year after the final surgery showed mild bladder deformity and bladder compliance of approximately 13 mL/cmH_2_O (Figure 4). It has been more than 2 years since the patient's final surgery. He has experienced no urinary incontinence or renal dysfunction and has not developed a febrile UTI.

## 3 Discussion

In this report, we present the case of a patient with NLUTD due to spina bifida who finally achieved urinary continence after undergoing a GMF for recanalization and fistula after BNC and urethral dissection. The noninterposition of any vascularized flap was a factor of failure in the initial BNC. Recanalization must be considered after BNC. In this case, urinary continence was eventually achieved using a GMF, which is often used in the repair of a urinary fistula.

Surgical management for neurogenic incontinence sometimes requires abandoning the native urethra due to intractable urinary incontinence, irreparable urethral erosion, or urethrocutaneous fistula. In these patients, BNC may be a last resort for providing urinary continence [1, 2]. In this patient, although the actual reason for urinary continence was unknown, we thought that the cuff of the night balloon had been accidentally inflated in the prostatic urethra, which destroyed the prostatic urethra and urethral sphincter, resulting in difficulty in catheterization and worsening urinary incontinence. We decided to close the bladder neck and create a continent catheterizable stoma to the umbilicus because self‐catheterization through the urethra was deemed difficult. We did not perform augmentation cystoplasty in this patient because there was no significant impairment of bladder storage function.

Recanalization or fistulas are frequent complications after BNC [2, 3]. The first choice of treatment is often an omental flap to cover the urethral stump. However, in this patient, the bladder and prostatic urethra were separated and closed without any intervention only because the great omentum to intervene was unavailable. When the great omentum is unavailable, other pedicled tissue flaps may be used, such as the rectus abdominis muscle flap, in accordance with previous reports [6, 7]. Smith et al. demonstrated that the rectus abdominis muscle flap is easily harvested and offers a well‐vascularized tissue for coverage of a bladder neck closure when an omental flap is not available [6]. We did not plan to use another pedicled tissue flap to intervene, which was a great failure in this case and probably induced the recanalization. There is no obvious answer to whether all patients should undergo any intervention; however, after BNC, a pedicled tissue flap to intervene should be performed.

The current case required a catheterizable stoma after BNC. Although the appendix is sometimes used as the catheterizable stoma, when the appendix is unavailable or unsuitable for use, the Monti ileovesicostomy is an excellent substitute for an appendicovesicostomy [5]. Furthermore, the spiral Monti procedure is a useful modification to the traditional Monti method because it allows for the creation of a longer channel when needed [5]. Because of its obstructed lumen, the patient's appendix could not be used. Thus, we applied the spiral Monti procedure because a somewhat long channel was required.

The gracilis muscle is a safe and reliable flap for various indications. Raup et al. reviewed GMF repairs for rectourinary and urinary cutaneous fistulae in patients who underwent pelvic radiation [8]. They found that the fistula was successfully repaired with GMF, although the radiated patients had a decreased quality of life. Sbizzera et al. also demonstrated an excellent success rate and low morbidity of transperineal repair with GMF for rectourethral fistula induced by localized prostate cancer treatment [4]. Michaud et al. reviewed patients with bladder and cloacal exstrophy who underwent BNC in combination with pedicle muscle flaps, including the rectus and GMF, to achieve urinary continence [7]. GMF was used to cover the re‐dissected urethra because a repeated transabdominal procedure using the rectus abdominis muscle flap was not feasible for this male patient. GMF is very effective in repairing urethral and perineal fistulas, as was shown in this patient.

## 4 Conclusions

We reported the case of a patient with spina bifida who underwent BNC for restoration of urinary continence. BNC is an effective last resort for intractable urinary incontinence. However, the possibility of recanalization or fistula after BNC must be kept in mind. Interposition and covering of the GMF are useful techniques for urethral and perineal fistulas.

## Consent

Written informed consent was obtained from the patient.

## Conflicts of Interest

The authors declare no conflicts of interest.
